# Physiologically-based pharmacokinetic simulations in pharmacotherapy: selection of the optimal administration route for exogenous melatonin

**DOI:** 10.5599/admet.625

**Published:** 2019-02-23

**Authors:** Adriana Savoca, Davide Manca

**Affiliations:** PSE-Lab, Process Systems Engineering Laboratory, Dipartimento di Chimica, Materiali e Ingegneria Chimica “Giulio Natta”, Politecnico di Milano, Piazza Leonardo da Vinci 32, 20133 Milano, Italy

**Keywords:** Melatonin, PBPK, administration route, transdermal, controlled release, simulation, clinical efficacy

## Abstract

The benefits of melatonin on human body are drawing increasing attention from several researchers in different fields. While its role as cure for sleep disturbances (e.g., jet lag, insomnia) is well documented and established, new functions in physiological and pathophysiological processes are emerging. To investigate these effects, there is need for the characterization of melatonin transport processes in the body and resulting pharmacokinetics. Although recent works propose physiologically-based pharmacokinetic modelling of melatonin, no work has yet highlighted the potential of PBPK simulations to shed light on melatonin pharmacokinetic aspects and discrimination among administration routes. This paper presents, validates, and discusses a versatile PBPK model featuring different ways of administration and compares the resulting pharmacokinetic profiles of intravenous, oral, and transdermal administration, with the goal of understanding which is the optimal route to achieve either physiological and/or supraphysiological melatonin levels.

## Introduction

In recent years, physiologically-based pharmacokinetic (PBPK) models have become widely-used and accepted tools to study, simulate, and predict drugs concentration in the body as well as provide insight on their pharmacological effects via combination with pharmacodynamic models. PBPK models are currently applied throughout the phases of drug discovery and development with various goals, *e.g.*, inter-species extrapolation, analysis of chemical toxicity or efficiency, investigation of different routes of administration, and study of inter-individual variability [1-5]. Indeed, PBPK simulations are extremely useful to study the pharmacokinetic differences among individuals, from pediatric patients [6] to healthy adults to *special* subjects, with particular conditions (*e.g.*, pregnancy [7]) or specific diseases with high probability of affecting drugs pharmacokinetics (*e.g.*, renal insufficiency or liver diseases). The reason is that PBPK models (first theorized in 1937 by Teorell [8]) incorporate the anatomy and physiology of the patients’ body into the mathematical description of drugs absorption, distribution, metabolism, and elimination (ADME) processes. Their recent success is also related to the current availability of modern tools to solve complex mathematical problems, such as systems of ordinary differential equations (ODEs) with a large number of parameters. *In silico* simulations are appealing because of the possibility to carry out “free” and fast experiments [9], compared to the actual clinical trials, whose costs and duration have increased over the past 20 years [10].

Not only drug discovery and development [11], but also the clinical practice may take advantage from simulation via PBPK models, as it tackles the problem of selecting the optimal dose that maximizes therapeutic efficacy while minimizing adverse effects. On one hand, inter-individual variability and medication errors are significant obstacles in this decision, on the other hand, the choice of the optimal administration route and dosing regimen are crucial degrees of freedom of this problem.

In this respect, melatonin is a useful and interesting case-study. The pleiotropic functions of melatonin in the human body are catalyzing the attention of several researchers in different fields, and its exogenous administration can follow different pathways. Although melatonin is particularly popular as a cure for sleep disturbances (*i.e.* jet-lag, insomnia), a number of other physiological and pathophysiological functions have been investigated and are still emerging. For instance, receptor-mediated actions include regulatory functions, *e.g.*, immune response, homeostasis, and blood pressure regulation [12-16]. Indeed, melatonin receptors are distributed in the whole body. Besides, non-receptor mediated actions are of great interest, especially the potency of its antioxidant, antiproliferative, and anti-inflammatory action via radical scavenging [17]. The application in chemotherapy in combination with other substances improves both the chances of survival and quality of the patients’ life [18].

In healthy people, melatonin is endogenously produced by the pineal gland. The production rhythm is entrained with the day-night cycle, with darkness causing the onset around 9-10 PM, peak between 2-4 AM (with *C*_max_ range 60-100 pg/mL), and baseline low values during the day at about 5-10 pg/mL [19]. However, this endogenous rhythm may be subject to either disruption or levels reduction, and medical doctors think that this has negative impact on the patients’ health status, especially in critically ills [20-22].

In order to identify the optimal melatonin dosage, a detailed characterization of exogenous melatonin ADME processes within the human body is recommended. Through the years, several authors have carried out pharmacokinetic studies to identify the most suitable dosage and route of administration to produce physiological and supraphysiological melatonin levels in different populations [23-26]. Although some recent works exist on the PBPK modelling of melatonin in the human body (*e.g.*, [27]), our aim is not only to provide a valuable PBPK model but also to compare melatonin levels that result from different routes of administration, *i.e.* intravenous (IV), oral (*per os*, PO), and transdermal (TD). The first goal is to understand which route has the highest potential to reproduce the endogenous profile of healthy patients, with the purpose of restoring melatonin physiological roles. The second goal is to identify the routes that allow achieving higher levels, with the purpose of producing pharmacological effects (for instance strong anti-oxidative action for ICU, intensive care unit, patients). Despite high inter-individual variability that is typical of melatonin pharmacokinetics (*e.g.*, related to different physical characteristics, genetic factors, and presence of impairments/diseases), we intend to show that *in silico* simulations can provide guidance and advice in selecting the optimal routes of administration and dosage, once the reliability of the employed model is verified. Indeed, model simulations constitute a powerful tool for optimal pharmacotherapy, especially in combination with experimental studies.

## Methods

In general, the PBPK approach combines anatomical and physiological aspects with mathematical modeling, by assuming that the organs and tissues of the human body can be represented by compartments with homogeneous concentration. The reference model of this work (from [28]) considers 8 compartments in the description of the human body: Plasma, Gastric Lumen (GL), Small Intestinal Lumen (SIL), Large Intestinal Lumen (LIL), Liver, Gastro-Intestinal Circulatory System (GICS), Poorly perfused Tissues (PT), and Highly perfused Organs (HO). Actually, some compartments represent single organs while other compartments represent lumped parts so to reduce the number of model parameters. In fact, a too high number of parameters may lead to mathematical predicaments of over-parameterization and model identification (see [10] for an exhaustive discussion on this topic). The HO compartment stands for organs that are highly perfused by blood, *i.e.* kidneys, lungs, spleen, and heart. The PT compartment lumps tissues that are poorly reached by blood vessels, *e.g.*, adipose tissue, skin, and muscles (specifically in ill/treated patients). The GICS compartment lumps the portal vein, the mesenteric artery, and the microcirculatory blood vessels of the gastrointestinal system.

We applied some modifications to this basic structure of the model to adapt it to melatonin pharmacokinetic features. Particularly, we added (i) the pineal gland, and (ii) the salivary glands. Pineal gland is the source of endogenous melatonin. Within our PBPK model, the material balance on the pineal gland accounts for the production of endogenous melatonin with a term that exhibits a 24-h periodicity (see [2]). Several authors evaluate melatonin endogenous and exogenous amount by measuring either saliva and plasma or only saliva concentrations [29-32]. Thus, we found more correct (from a physiological point of view) to add the salivary glands to the model compartments. The drug material balances, in the form of an ODE system, describe the concentration dynamics of melatonin in each compartment. Finally, an additional equation allows accounting for the dynamics of melatonin main metabolite 6-sulfatoxymelatonin (aMT6s).

In case of IV route, the drug directly inputs the Plasma compartment. Conversely, in case of PO administration, the drug enters the GL and moves through SIL and LIL to be absorbed through the intestinal walls and conveyed to Liver via the portal vein. This results into the so called “first-pass metabolism effect”. After that, it is drained from the Liver to reach the systemic circulation and distributes to the other organs and tissues via the bloodstream. It is worth stressing that the model structure takes into consideration GL, SIL, and LIL only in case of PO administration. In fact, in other cases, we assume that the drug counter-diffusion from GICS to SIL and LIL is negligible, and therefore it is possible to neglect such compartments, along with GL and reduce significantly the number of ODEs. We do not report here the complete mathematical description of the model, as it is extensively detailed in [10,28].

While in case of IV and PO routes, the skin is incorporated into the PT compartment, in case of TD administration the skin becomes the mean for drug absorption and therefore calls for a specific and detailed description. In particular, melatonin evolution has to be considered not only in time but also along the skin depth coordinate. Thus, the homogenous approach (based on the perfectly mixed hypothesis) to compartment modeling is replaced and the resulting skin mathematical description involves partial differential equations (PDEs) with suitable boundary conditions [2]. Particularly, three skin layers are considered: (i) stratum corneum that is the most external and thinnest but also the main barrier, (ii) viable epidermis that may constitute a metabolism site, and (iii) dermis, from which the drug is supposed to reach the systemic circulation via the contained blood vessels, and then distribute to the rest of the body.

In case of TD administration, the PDEs describing the skin and the ODEs describing the rest of the body are combined via the finite differences method. In fact, the PDEs are discretized respect to the spatial coordinate (*i.e.* skin depth) and therefore converted to ODEs [2].

Independently of the administration pathway, the model parameters can be divided into three categories: (i) individualized, (ii) assigned, and (iii) regressed. Individualized parameters (*e.g.*, volumes of compartments and flowrates among them) are calculated according to empirical correlations that are available in the literature and depend on the patients’ physical characteristics. We consider as specific features the sex, body weight, and height. Assigned parameters are some drug physicochemical properties whose value can be determined from the scientific literature (*e.g.*, protein binding). Some parameters, strictly related to the transport properties, can be neither found in the literature nor calculated by empirical correlations (*e.g.*, diffusivity, transfer coefficients, metabolic constants), thus they are obtained via a non-linear regression procedure respect to experimental data from the literature. Indeed, although the value of some transfer coefficients might be determined from *in vitro* studies, such experiments would not account for the interactions among organs and tissues in the living organism, and therefore would affect the reliability of the mathematical model and consistency/validity of the simulated results.

Once the model transfer coefficients and metabolic constants are identified (with data from [33] for IV route, [34] and [29] for PO, and [35] for TD), a model validation with additional experimental pharmacokinetic data allows assessing its prediction capability. To do so, we chose (i) the median squared error (MeSE) (Eq. (1)) over the mean squared error (MSE) [27] for robustness reasons, (ii) the difference between the experimental area under the curve AUC_exp_ and the model prediction AUC_mod_ ([28], Eq. (2)), and (iii) the difference between the observed and predicted values of *C*_max_. Comments on the difference between the observed and predicted values of *T*_max_ are also present. The AUC is calculated via trapezoidal rule over the NM measured concentration values. We consider satisfactory MeSE values below 0.1 [ng/mL] and %Δ_AUC_ values below 30 %.


(1)

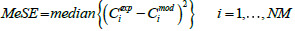




(2)

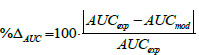



Once the prediction capability of the PBPK model is evaluated, it is interesting to assess *in silico* the optimal administration route. In particular, we investigated three distinct administration routes: (i) IV continuous infusion over 7 h, (ii) PO in both the immediate and controlled release (CR) formulations (the last one with a release time of 7 h), and (iii) TD with a standard patch of 10 cm^2^. The PO (CR) tablet release is modeled according to the dissolution characteristics elucidated in [36] and employed in [35]. Results from all the administration routes are compared for an assigned dose range between 0.75-12 mg, grounding on the state-of-art pharmacokinetic studies on exogenous melatonin administration. For this preliminary study, we do not consider high doses [37]. Our virtual subject is a healthy adult male of 80 kg and 185 cm. To provide a quantitative comparison of the pharmacokinetics resulting from the three administration routes, we calculate and compare the AUC and the maximum concentration *C*_max_. Finally, we comment on the concentration dynamics in the different compartments of the body that result by simulating the administration of melatonin 3 mg via IV, PO, and TD routes to the same *in silico* patient. For an unbiased comparison of melatonin ADME, we intentionally neglected the endogenous melatonin production.

## Results and Discussion

We computed the prediction performance with data coming from melatonin pharmacokinetic studies. The validation cases for each route of administration are proposed and discussed.

### IV validation case

**Figure 1:**
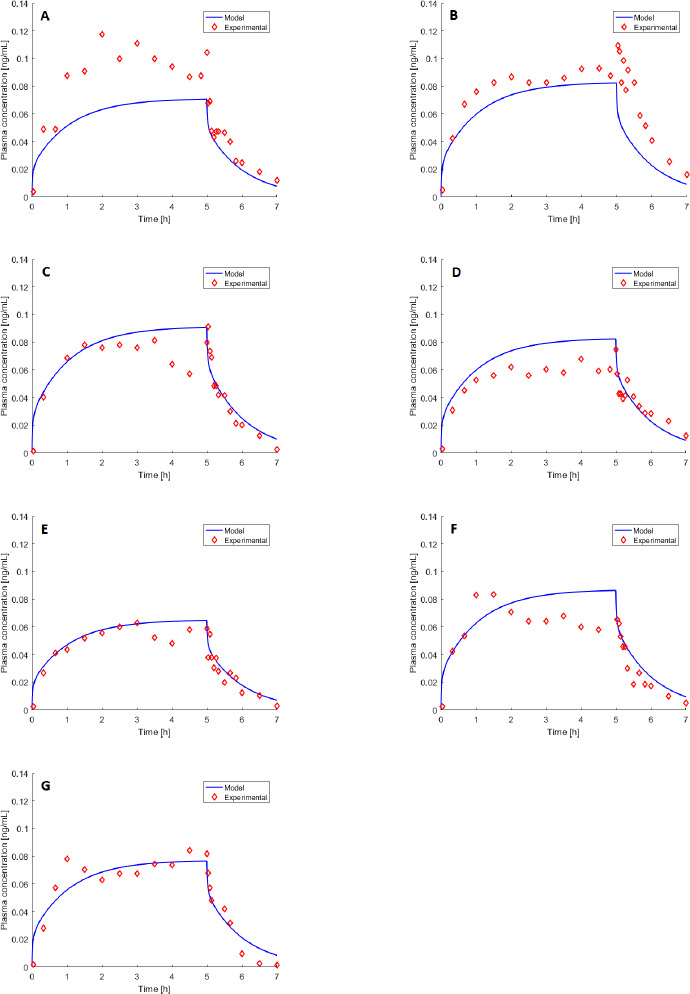
- Experimental data (red diamonds, [37]) represent the pharmacokinetics of 6 healthy subjects (A-F panels) and 1 pinealectomized patient (G panel) who received IV 20 μg infused over 5 h. The blue continuous line is the model-simulated pharmacokinetic profile.

**Figure 2:**
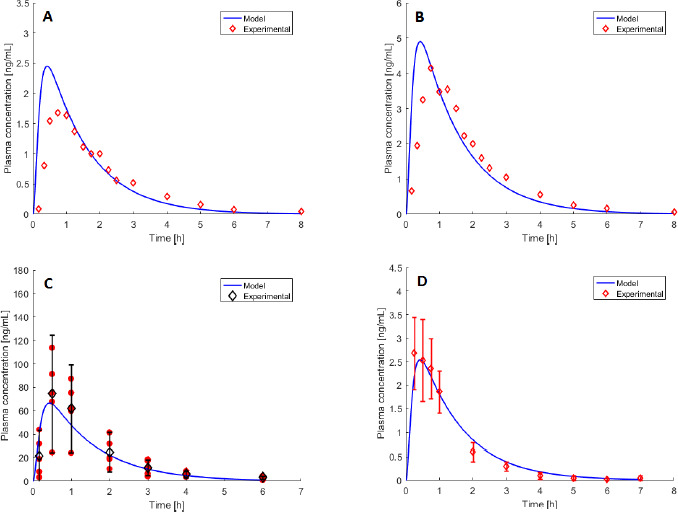
- A-B panels: experimental data (red diamonds, [33]), represent the average pharmacokinetics of 12 subjects administered with melatonin PO 2 and 4 mg. C panel: experimental individual (red dots) and median (black diamonds) pharmacokinetics after melatonin PO 50 mg [25]. D panel: experimental data (red diamonds) averaged over 5 healthy subjects from [40] (melatonin PO 2 mg). The blue continuous line is the model-simulated pharmacokinetic profile.

**Figure 3:**
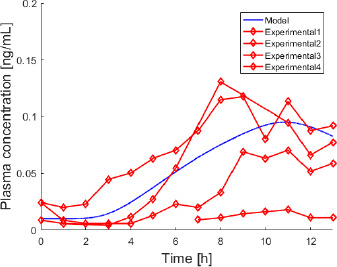
- Experimental data (red diamonds, [41]) are the individual pharmacokinetic trends, resulting from melatonin TD 2.1 mg over 12 cm^2^ patch administration. The blue continuous line is the model-simulated pharmacokinetic profile of the averaged subject.

Figure 1 shows the model curve resulting from the simulation of 20 μg IV infusion over 5 h to 6 healthy subjects (A-F panels) and 1 individual subjected to pinealectomy 2 years earlier (G panel), as in [39]. Experimental data (red diamonds) show the individual pharmacokinetic profiles. The model performance (*i.e.* the blue line) is acceptable, but for A panel of Figure 1. Nevertheless, the values of the performance indexes (see Table 1) remain quite satisfactory, as even the AUC value of that patient (A panel) is only slightly higher than 30 %. It is worth observing that the experimental inter-individual variability of melatonin levels is reduced in case of IV administration if compared to other routes (see also Figure 2 and Figure 3). As a result, also confidence intervals of the IV model parameters are narrower (see values Tables A-C reported in Appendix). Figure 1 (G panel) shows the experimental trend for the pinealectomized patient where the model performance is as good as for the others. The IV model was further tested with experimental results of additional patients from the same study (subjected to bolus injection) and supplementary validation cases [34,39], for which the results of the %Δ_AUC_, %Δ*C*_max_, and MeSE (not reported) are adequate as well. In this case, we do not calculate the experimental/predicted *T*_max_ and the relative error, because in case of IV constant rate of infusion the *T*_max_ corresponds to the infusion duration (*i.e.* in this case study, equal to 5 h).

### PO validation case

Figure 2 shows experimental results on melatonin concentration in case of PO administration. The blue line in Figure 2 (A and B panels) simulates the pharmacokinetics after administration of 2 and 4 mg respectively, to 12 healthy volunteers [33]. The experimental data (red diamonds) show mean concentration values of the volunteers group. Figure 2 (C panel) shows both individual (red dots) and median (black diamonds) concentration profiles of 5 healthy subjects administered with 50 mg [25]. Finally, Figure 2 (D panel) shows the simulation (blue line) of the averaged profile of 5 healthy subjects administered with 2 mg [40]. In all these cases, only a single curve is displayed, because the literature data report only averaged demographic and/or pharmacokinetic data. Despite the literature differences in features and dosages, the model performance is acceptable as the simulation curve is near to the average experimental profile in all the cases. In fact, Table 2 lists low values of MeSE, except for Figure 2 (C panel).

It is worth observing that the simulated profile (*i.e.* blue line) anticipates the experimental data (see Figure 2 (A, B, and C panels)). The difference between the observed and predicted *T*_max_ is about 30 min. This may be related to digestion features and to the patients’ condition (*e.g.*, fed or fasting). Future work should adapt the PO model to such issues. However, the observed *T*_max_ depends also on the experimental protocol, and in particular, on the blood sampling time. In addition, this parameter is affected by a certain degree of experimental error. The %Δ_AUC_ in Table 2 is always below 15 % while the relative error between the observed and predicted *C*_max_ is below 15 % except for case A. It is fair to acknowledge that the pharmacokinetics resulting from the PO route features a higher degree of inter- and intra-individual variability compared to the IV route, because of several interacting factors that affect absorption (*e.g.*, pH, stomach emptying time, intestinal transit times, and variation of blood supply to stomach and intestine) and metabolism (*e.g.*, genetic factors and presence of diseases). In fact, MeSE results for the IV validation cases are at least one order of magnitude lower and, consistently, confidence intervals of the model parameters are larger (Table B in Appendix). Additional case studies are employed for validation, with similar results in terms of performance assessment [39].

### TD validation case

Figure 3 shows a validation case from [41] for the TD route. In the study, melatonin was administered 2.1 mg/12 cm^2^ as TD patch. Since the reported demographic data consist only of averaged measures over the subjects’ group, the model curve (blue line) is the pharmacokinetics of an averaged individual, while red diamonds represent the experimental values of melatonin concentration of the individuals who took part to the study, connected with red lines for the sake of clarity. The model prediction is quite near to three out of four individual trends. The most distant individual trend shows atypical pharmacological levels, which may be related to either differences in the skin features of that specific subject and/or melatonin dermal deposition [41]. This results into a nonsensical value of %Δ_AUC_ (Table 3). In general, TD pharmacokinetic data show high inter-individual variability having to do with the process of transdermal absorption [41]. This aspect is also reflected in the confidence intervals of the model parameters (Table C in Appendix) and in the variability of observed *C*_max_ and *T*_max_ values, although it should be remarked that blood sampling occurred every hour. Thus, it is not guaranteed that the real experimental maximum value corresponds to the observed *C*_max_. In any case, the %Δ*C*_max_ is around 25-30 % for the first three individuals. As far as the *T*_max_ is concerned, it is worth observing that the model seems to predict a slower absorption compared to the experimental trend. Despite the high values of %Δ_AUC_, it is likely that the reliability of the model for this route might further improve by relying on a higher number of experimental data sets for the identification of the parameters.

For all the considered routes (*i.e.* IV, PO, and TD), the results are acceptable enough to continue with the analysis of melatonin ADME in the body as a function of the different administration pathways.

### In silico simulations for optimal dose selection

**Figure 4:**
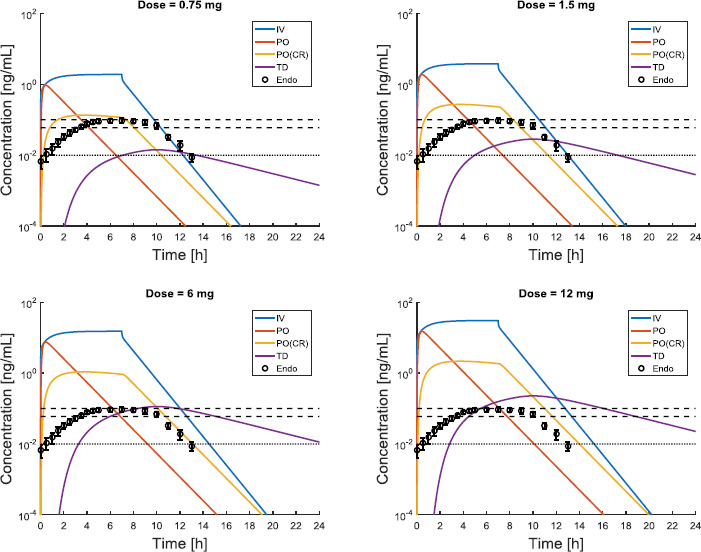
- Dynamics of the melatonin plasma concentration over 24 h after IV, PO, PO (CR), and TD administration of 1.5 to 12 mg to a virtual subject (male, adult, 80 kg, 185 cm). Black circles describe the endogenous profile in healthy adult volunteers [32]. Black horizontal dashed lines indicate the range of endogenous *C*_max_ in healthy subjects (60-100 pg/mL). Black dotted line marks the average value of daily melatonin baseline in plasma.

A number of pharmacokinetic studies focuses on selecting the optimal dose that produces either physiological (*e.g.*, [38]) or supraphysiological levels. In fact, while physiological levels can improve sleep maintenance and resynchronize circadian rhythms [17], supraphysiological levels may produce strong antioxidant action [25] and analgesic effects [37]. To investigate melatonin pharmacokinetic properties, a few studies compare the *in vivo* results of different routes of administration [34], and/or specific populations (*e.g.*, elderly [26], critically ills [24], patients suffering from severe oxidative stress [25]). To prove the efficiency of *in silico* simulations within this context, we compare the pharmacokinetic profile resulting from three different routes, with doses ranging from 0.75 to 12 mg. The selected range is considered safe as it has been covered by a number of pharmacokinetic studies. Figure 4 shows the results of the simulations, along with comparison to experimental data of endogenous profile in healthy adult volunteers from [32].

**Figure 5:**
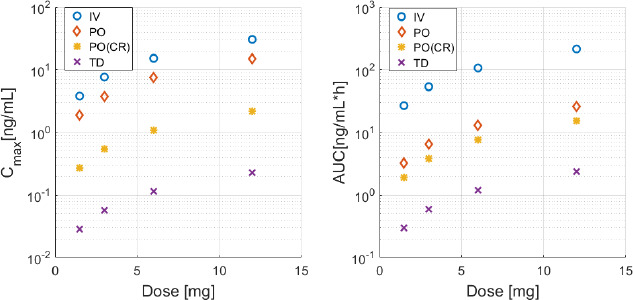
- Comparison of pharmacokinetic parameters *C*_max_ (left panel) and AUC (right panel) resulting from the three routes of administration. The x-axis reports the simulated dose range, *i.e.* 0.75 to 12 mg.

As expected, smoother and more sustained levels are achieved via TD and PO (CR) formulations. The slow absorption phase, which is characteristic of TD release, proves particularly suitable for mimicking the endogenous levels produced by the pineal gland. Equally, the PO (CR) solution provides sustained levels as well (*T*_max_ about 4 h), coupled with a steeper absorption (see especially the case of 0.75 mg). This difference in the velocity of absorption has to be considered in the choice of the administration time, as this will affect the onset time of melatonin effects. In case of PO (CR) 0.75 mg administration, shifting the time of administration would allow quite a close imitation of the endogenous profile. The same consideration holds for the case of TD 6 mg administration. Thus, not only dosing, but also the time of administration is a key degree of freedom in the problem of melatonin delivery optimization to restore/produce physiological levels. Failing in considering this aspect would likely result into unsatisfactory outcomes in terms of pharmacodynamic effects. In this sense, PBPK simulations can be used as a tool for therapy design, to determine the time of administration that more likely leads to the desired effects. It should also be noted that, although the TD route produces sustained levels over 24 h, it is unlikely that the subject will manifest adverse effects, for instance related to sleep. Firstly, levels are quite similar to the endogenous pattern (see the black circles), and after about 10 h, they start decreasing towards the daily baseline (black dotted line). Secondly, doses up to 3500 mg (PO) have been administered without any acute adverse effects and the scientific literature does not report any toxic threshold for melatonin dose [37]. For instance, in [37] there is no evidence of sedative effects for doses up to 100 mg (IV), which would produce more than 3-order-of-magnitude higher levels than those shown in Figure 4, case 12 mg via TD route (according to our simulations and consistently with experimental results reported in the study). Predictably, even low doses of continuous IV infusion produce the highest levels and bioavailability, thus it is probably the most appropriate mean to reach prompt pharmacological (*i.e.* supraphysiological) levels. In fact, even for the lowest dose considered (*i.e.* 0.75 mg), the resulting plasma concentration is an order of magnitude higher than the endogenous one (see black circles compared to the blue line). On the contrary, TD administration should be excluded for the purpose of producing pharmacological levels (see highest doses 12 mg in Figure 4 and *C*_max_ value in Figure 5). Figure 4 also shows that in case of melatonin, PO immediate release formulation is not able to produce sustained levels. However, for doses higher than 5 mg, this administration route can be considered to reach pharmacological levels, alternatively to IV infusion. All of these considerations are confirmed by the values of the pharmacokinetic parameters AUC and *C*_max_, compared in Figure 5. The highest AUC is in fact associated with the IV continuous infusion route, whereas the other routes of administration produce lower values. Another possibility to be explored is the combination of oral immediate release and CR formulations.

Depending on the treatment goal, for instance the attainment of either endogenous or pharmacological levels, it is possible to explore additional contributing factors, other than the route, dose, and time of administration. In fact, different dissolution curves can be employed in the PO (CR) formulations and can be combined with the PBPK model to study the resulting ADME processes. The same approach can be applied in case of TD route, since both the features, position, and application extent of the patch are degrees of freedom for the medical doctor. The main degree of freedom of the IV infusion route is its duration. Once the main goal is assigned (in terms of ideal pharmacokinetic profile for a specific application), an optimization can be carried out to identify the optimal dose and dosing regimen by considering those additional degrees of freedom.

**Figure 6:**
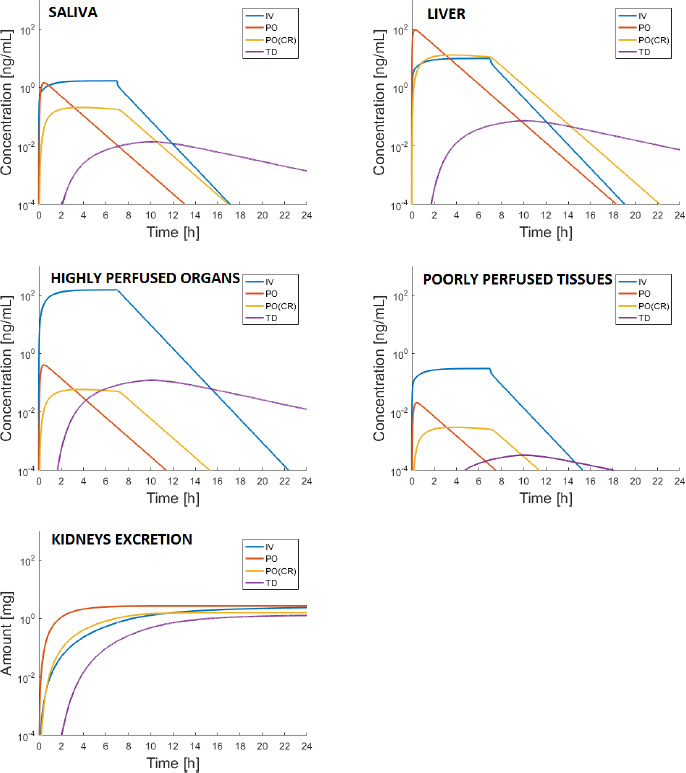
- Simulation of the melatonin concentration dynamics in the compartments Saliva, Liver, Highly and Poorly perfused Organs/Tissues, and the Kidneys-excreted amount after administration of 3 mg via IV, PO, PO (CR), and TD.

Since melatonin roles affect several organs and tissues, with cerebral, immune, gastrointestinal, cardiovascular, renal, and endocrine functions [12], and melatonin receptors are distributed in the whole body, model compartment levels should be visualized and discussed, as well. Figure 6 shows the simulation in different compartments for 3 mg administered IV, PO, PO (CR), and TD. The slow drug absorption, typical of TD administration, is reflected in the slow distribution to the organs/tissues of the body. Conversely, IV infusion induces higher levels in all the compartments (see Highly perfused Organs and Liver compartments in Figure 6). Thus, in case the goal of melatonin administration is a diffused anti-oxidant action in the patient body via radicals scavenging, this route should be preferred. The same can be stated in case of immune system enhancement (hence with potential beneficial effects in terms of cancer cells detection and elimination). In addition, when target organs are the highly perfused ones (*e.g.*, pancreas, liver, and kidneys), this route should be definitely considered. When a more localized target action is required, it should be considered that in case of PO administration, higher levels are expected in the liver and gastrointestinal tract, as confirmed by the model simulation. According to [14], melatonin is gastro-protective at endogenous levels, whereas pharmacological levels of melatonin (in combination with other drugs) contribute to healing of gastroduodenal ulcers. The difference of goal (i.e. gastro-protection vs healing of local ulcer) will be the discriminating factor for selecting the most suitable dose to produce either endogenous or higher levels. The velocity of excretion via the kidneys is comparable for both the IV and PO routes, and is faster for these routes when compared to TD. Concluding, anatomical and physiological considerations can be converted into quantitative data to be carefully assessed, analyzed, and visualized via PBPK model simulations. This kind of information is not only useful when several routes of administration are viable, but also especially important when the drug target site is not plasma.

## Conclusions

While PBPK simulators allow evaluating melatonin levels in plasma and the rest of the body, further practical considerations should support the pharmacokinetic investigation with the aim of achieving optimal clinical efficacy. In fact, while IV route may hold the advantage of the highest bioavailability and fastest distribution to organs and tissues, most of the patients may find it distressing. Therefore, it may result suitable only for specific categories such as critically ill patients, who usually receive continuous infusion of different drugs and enteral nutrition for quite long periods. On one hand, PO route is easy and simple but it is subject to first-pass hepatic metabolism, which implies a certain degree of inter-individual variability related to different metabolism characteristics, and different patients’ features (*e.g.*, gastrointestinal pH, temperature, and other previously mentioned factors). As well as PO (CR) option, TD route allows obtaining sustained levels and avoids first-pass hepatic metabolism. On the other hand, it is subject to slow absorption through skin, possible metabolism within viable epidermis, and high inter- and intra-individual variability related to different skin features.

In this work, we introduced and discussed a case-study to compare pharmacokinetic levels resulting from different doses and three administration routes, *i.e.* IV, PO, and TD, also considering both oral immediate release and CR formulations. PBPK simulations are particularly interesting for their intrinsic nature and structure, because they provide quantitative information on the drug ADME processes in the body. Besides, coupling with intelligent drug design and *in vitro* experiments enhances the potential to maximize their efficacy. As far as melatonin is concerned and with reference to both practical and pharmacokinetic aspects, it is possible to conclude that PO (CR) and TD routes represent the best options in case of disruption of the endogenous rhythm (*e.g.*, in people suffering from either insomnia or jet-lag and critically ills). Equally, PO (with doses significantly higher than 3 mg) and IV infusion are preferable when higher concentration levels are required for other goals, for instance to contrast severe oxidative stress and possibly cancer, and target specific organs as sites of pharmacological action. This work can be extended and improved by focusing on one administration route and running a numerical optimization of the melatonin dose respect to a target trajectory, also considering a number of degrees of freedom depending on the selected route. It is also worth stressing the transferability of the presented approach to any other drugs that are versatile from the point of view of the administration routes. Such investigations may become especially interesting in case of drugs with narrow therapeutic windows, such as chemotherapy drugs whose pharmacokinetics quantification is essential and critical.

## Figures and Tables

**Table 1:** - Performance indexes %Δ_AUC_, %Δ*C*_max_, and MeSE values for the IV validation case.

Patient	%Δ_*AUC*_	%ΔC_*max*_	*MeSE*
**A**	31.2	32.1	2.4e-4
**B**	18.8	24.5	2.6e-4
**C**	14.2	13.4	4.6e-5
**D**	20.6	10.1	8.3e-5
**E**	10.5	10.4	2.0e-5
**F**	16.3	31.8	4.5e-5
**G**	0.7	6.4	5.1e-5

**Table 2:** - Performance indexes %Δ_AUC_ and MeSE values for the PO validation case.

Panel	%Δ_*AUC*_	%ΔC_*max*_	*MeSE*
**A**	2.6	40.1	0.009
**B**	7.3	13.5	0.103
**C**	14.3	10.7	5.68
**D**	8.4	5.0	0.004

**Table 3:** - Performance indexes %Δ_AUC_, %Δ*C*_max_, and MeSE values for the TD validation case.

Patient	%Δ_*AUC*_	%ΔC_*max*_	*MeSE*
**1**	39.1	34.9	8.2e-4
**2**	38.1	27.7	2.1e-4
**3**	26.2	23.6	5.4e-5
**4**	>100	>100	4.0e-3

## Appendix

**Table A.** – Key model parameters in case of the IV route, correlated by 90 % confidence intervals.

Parameters	Description	Regressed values	90 % *CI_lb_*	90 % *CI_ub_*
***k_T-P_* [ *min^−1^*]**	Plasma-poor tissues transfer coefficient	0.3955	0.373	0.418
***k_P-T_* [ *min^−1^*]**	Poor-tissues plasma transfer coefficient	0.8	0.774	0.826
***k_HP-P_* [ *min^−1^*]**	Highly perfused organs- plasma transfer coefficient	0.047	0.045	0.05
***k_P-HP_* [ *min^−1^*]**	Plasma-highly perfused organs transfer coefficient	1.48	1.416	1.544

**Table B.** – Key model parameters in case of the PO route, correlated by 90 % confidence intervals.

Parameters	Description	Regressed values	90 % *CI_lb_*	90 % *CI_ub_*
***k_A, SIL_* [ *min^−1^*]**	*SIL* absorption constant	2.205	0.245	5.655
***k_CA, SIL_* [ *min^−1^*]**	*SIL* counter-diffusion constant	2.920	0.158	5.683
***k_A,LIL_* [ *min^−1^*]**	*LIL* absorption constant	0.167	0.003	0.337
***k_CA, LIL_* [ *min^−1^*]**	*LIL* counter-diffusion constant	0.455	0.059	0.851
***Eff_H_* [ *−*]**	Hepatic metabolism efficiency	0.467	0.235	0.699
***Eff_K_* [ *−*]**	Kidneys excretion efficiency	0.053	0.007	0.113

**Table C.** – Key model parameters in case of the TD route, correlated by 90 % confidence intervals.

Parameters	Description	Regressed values	90 % *CI_lb_*	90 % *CI_ub_*
	*SC* Diffusivity	3.352*10^−5^	1.269*10^−5^	5.431*10^−5^
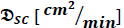
	*VE* Diffusivity	5.943*10^−3^	2.122*10^−3^	9.763*10^−3^
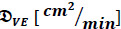
	*DE* Diffusivity	2.776*10^−3^	1.395*10^−3^	6.946*10^−3^
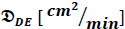
***k_part1_* [*−*]**	*SC/VE* Partition coefficient	1.763	1.209	2.317
